# Sexual practices of HIV-positive individuals attending antiretroviral treatment (ART) in Addis Ababa public hospitals: findings from in-depth interview

**Published:** 2012-12-17

**Authors:** Yadeta Dessie, Merga Deresa

**Affiliations:** 1 Department of Public Health, Colleges of Health and Medical Sciences, Haramaya University, Harar, Ethiopia; 2 School of Nursing and Midwifery, Colleges of Health and Medical Sciences, Haramaya University, Harar, Ethiopia

**Keywords:** Sexual practices, HIV-positive, people living with HIV, antiretroviral treatment

## Abstract

**Introduction:**

The rollout of Antiretroviral Treatment (ART) and improved health care services contributed in recuperating the quality of life and the functional status of HIV-positive people. These clinical effects of the treatment and cares are believed to bring a change on their sexual practices. The objective of this study was to explore the sexual practices of the HIV-positive people who were getting ART in selected Addis Ababa public hospitals.

**Methods:**

A qualitative in-depth interview was conducted. The interviews were made by trained nurse counselors of the same sex and were tape recorded. Verbatim transcription was made before the analysis. Thematic categorizations were made to present the findings.

**Results:**

Most participants expressed regained sexual desires with initiation of ART while some others didn't appreciate the regains. Not using condoms or inconsistently using them was identified risky sexual practices. Sero-discordances and sero-status non-disclosure were common issues among the partners.

**Conclusion:**

Sero-status non-disclosure, non-use of condom and inconsistent using them were common sexual issues. These hinder the efforts that are being made to reduce new HIV infections and re-infections. Interventions against these problems can be made when clients come for their ART treatment and clinical care follow up.

## Introduction

The advancement in medical management has been prolonging the life and improving the health status of the people infected with HIV [1, 2]. For example, the implementation of ART has improved the physiological, social, and psychological make ups of these individuals [3–5]. However, these affirmative gains in turn are likely to increase the sexual activities of those whose illness had previously inhibited the behaviors [3, 4].

Many studies have found out that, after the initiation of ART, HIV-positives individuals are becoming sexually active [1, 2, 6–9]. For those who are sexually active, safer sex practice is recommend to prevent further infection transmission and the chance of re-infection with new strains of HIV virus [10]. In this regard, heterogeneous findings were reported. Many studies reported that many HIV-infected individuals reduced risky sexual practices though substantial numbers, ten to sixty percent continued to engage in HIV transmission-risk behaviors. These likely expose them to re-infection with drug resistant HIV strains and further infection with other Sexually Transmitted Infections (STI) which in turn fastens the AIDS progression [2, 3, 5, 11, 12]. There was also a report that indicated the initiation of ART results in an increased risk of sexual practices [13].

From the previous studies common factors that attributes to the risky sexual practices were the type of partner relationship, psychological conditions, and behavioral related factors [1, 8, 14, 15]. These factors are so inconsistent and not remain the same under different circumstances. Importantly, in the era of scaling up of the ART to achieve the universal accessibility of treatment, this is the area of concern. To devise effective strategies and programs that would protect both people living with HIV their sexual partner and controlling further spread of the virus, exploring the sexual practices among these population in general and causes of risk sexual practice in particular is fundamental. With these premises, our current study was aimed at exploring what are the sexual practices of those HIV-Positive individuals taking ART seems with the emphasis on the risky sexual practices. We also looked at the common reasons for not using condoms, partner relationship, and the perception toward condom use.

## Methods

### Study setting

A qualitative cross-sectional in-depth interview was conducted with ART taking individuals from four selected public hospitals in Addis Ababa, Ethiopia, in March 2009. In the city, there were 9 public hospitals, 23 public health centers and 21 public clinics in 2008. Then there were 222,828 (7.5%) HIV positive people [16] and during the study period, 35,207 People Living With HIV (PLWH) were attending ART [17].

### Participants

The study participants were HIV positive people who were coming to the hospitals for their ART and follow-up care. Those who were 18 or above years of age and who were sexually active in the last three months prior to the study were included in the study. They were approached by the ART counselors who had been working in the respective hospitals. They were requested to participate on the study after explaining all the necessary information and their informed consents were taken based on the approved protocol. Thirteen participants were selected and interviewed.

### Data collection

The question items in the in-depth interview were socio-demographic (age in completed years, sex, marital status, current occupation, religion and highest educational level), sexual practices, and partners' relationship. The themes on sexual behavior included the sexual practices before and after being infected with HIV and the practice of using condom in the last three months. The items on sexual partner referred to the kind of sexual partner, to disclosure of sero-status to partner, and to the kind of responses received from the partners. The interview was conducted in Amharic language by trained nurse counselors of the same gender, and the investigator was involved only in note taking. The interview was tape recorded with the consent of the participants.

### Data analysis

The, audio-record was carefully transcribed in verbatim by the investigator and then translated into English. After the translated version had been read critically, analysis was made by categorization, narration and description. Results were presented in the form of theme that includes life time sexual partner and condom use before testing positive, ART and sexual desire, respondents sexual partner sero-status and condom use in the three months prior to the study, importance of condoms after testing positive, and reasons for HIV-positive people do not use condoms were explored.

### Ethical considerations

Ethical clearance was secured from the Addis Ababa University Institutional Review Board (IRB). Before any procedure conducted respondents were explained of the purposes, procedures, risks and benefits, and the private and confidential nature of the study. Participation was volunteer and declining for participation would not have any negative consequences in terms of service provision to them. Written informed consent was obtained from each respondent before commencement interview.

## Results

### Socio demographic and treatment characteristics of the participants

The in-depth interview was conducted with thirteen purposively sampled ART attendees. The mean age of the participants was 32.3 ± 5.0 (SD). Three of them were above 35 years of age, while the rest were from 25-35 years. Eight of the respondents were female, nine were married, and one was widowed. Most of the respondents (84.6%) had completed Grades 7-12. Two were Muslim, two were Protestant, and the rest were Orthodox in their religion. Seven of the respondents were unemployed. The latest respondent diagnosed four months ago, while the earliest did before seven years with the mean of 31.5 months. The participants had been on ART on average for 21 months.

### Partner relationship and condom utilization before diagnosis

Almost all of the study subjects (92.3%) reported that they had practiced sex with more than two partners before they were proved HIV-positive. While some of them had used condom irregularly (N=8), the rest had never used it then.

A 28-year-old, unmarried lady interviewee explained: *"before I came to know my sero-statsus as HIV positive, I was working as a bar lady. I had many partners, but two of them were regular partners, and I didn't use condom with them considering them as my true lover, but I used condom with all the rest partners I had"*.

A 30-year-old married lady narrated her sexual behavior and life partner before being HIV positive as: *"Before I come to know my HIV status, I had two partners including my current husband, where the first one was my boy friend and he was the first person with whom I had a sex for the first time in my life. Because, I love him very much, most of the time we had sex without condom. Later, I heard from my friends as he also had another partner besides me and I have stopped the relation I had with him. With my current partner(husband), we used to use condom at the beginning, but later not"*.

A 33-year-old, unmarried man said: *"Because I was young, I was with many partners and condoms were unappealing for me that I haven't used them in most of the sexual intercourses I had"*.

### Antiretroviral Treatment (ART) and sexual desire

After they had been diagnosed positive and begun the ART, half of them felt an increase in their sexual desire. The rest, responded as their desire to have sex have been depressed since testing positive and even after initiating ART, and there were no-significant change with their sexual desires.

A 34-year-old, grade 4, married man explained *"Before I knew my sero-status, I was frequently sick and I didn't feel happy and so was my sexual desire. However, thanks to the drug now, I am feeling healthy and my desire to have sex is very nice and I regained it"* A 29-year-old lady said *"My sexual desire has been depressed since the date I knew my sero-status...sexual desire is associated with internal health and conveniences. But I was having sex only to keep the interest of my husband."*

### Sero-status of the respondents' sexual partners and condom utilization in the last three months prior to the study

Ten of the sexual partners of the respondents were HIV-positive. In the last three months, the respondents had been using condom consistently (61.5%) and inconsistently (15.3%), while some did not use it at all (23.1%).All of the respondents reported that they had been with only one sexual partner in the last three months. A majority of them were with their wives and few were with a partner they considered as steady (regular).

### Responses from those who were consistently using condoms

A 36-year-old-man who was tested positive 3 years before and was living with his sero-negative partner said: *"My partner was sero-negative as we tested together last year and I have the responsibility to take care of her at each of our sexual act we used condom...even in case she is positive, it is our life. Because, drug adaptation means resistance can occur as the viruses are different in different individuals and I will continue to use the condom consistently"*.

Another 38-year-old married women living with her HIV positive partner said: *"My husband is a truck driver, he comes once or twice in a month and each time we had sex we used condoms, because he is the prime person in supporting our life, I should care for him, as there can be drug adaptation of the virus if we don't use condom"*.

Yet another 28-year-old unmarried lady said: *"We both work in one faith-based organization and he was the one who helped me in order to get employment there. He asked me for partnership and I disclosed myself as I am HIV positive and he told me that he is negative, but he accepted me. Even if we used condom consistently, I am not sure for he is negative as he feels free while having kiss with me"*.

### Responses from those who were not using condom

A 32 year-old married lady stated that: *"I had sex only with my husband; we had it once in two weeks and that were without condom. He told me that he is sero-negative even though not voluntary to be tested with me, but he didn't want to use condom. When I requested him to use condom, his reaction was not good and I felt bad with his reaction as it is me who is positive. He explained me that his blood and mine is quite different, so that the Virus cannot infect him, then I decided after all not to raise the issue of condom use with him" Another 25-year-old-married lady responded: "Similar to me, my husband is also positive and we didn't use condom, even before that (means before three months). If I had sex with someone else, I should use condom because I don't want to affect other people but now both of us are positive"*.

### Responses from those who used condom inconsistently

A 39-year-old man who was recently diagnosed HIV positive said *"I felt fear to tell my partner to use condom on the first day of sexual intercourse after I tested positive and had sex without condom. But on next, I disclosed myself and she was also tested positive, and since then we have been using condoms regularly"*.

### Respondents' response to the importance of using condom after testing positive

The interviewee raised different views on the importance of condom use after testing positive in the context of positive and negative partners. All of them stated that condom is important in protecting against unwanted pregnancy. Ten respondents mentioned the use of condom in relation to the risk of reinfection, risk of transmission and drug adaptation (wanted to say drug resistance). Seven also explained condom is important in preventing other sexually transmitted diseases. One respondent explained that he was counseled by doctors that having sex without condom is not good for his health and he was using condom consistently but was not able to clearly specify what problem unprotected sex might bring on them(with his partner) other than protecting from unwanted pregnancy.

A 33-year-old married lady expressed that: *The virus in me may be different from the virus in my husband, because I am taking the drug, but he is not, so this can bring problem on us if we have sex without condom"*.

Two of the respondents did not know the importance of using condoms, apart from protecting against pregnancy; both of them were living with their respective HIV positive partners. One 33-year old man explained: *"I know Condom is important to prevent pregnancy for that my wife is using other family planning, and I don't know for other use of condom since both of us positive"*.

### Views on why HIV-positive people fail to use condom

Four thematic points were raised concerning why HIV-positives fail to use condom: reduction of sexual pleasure use, fear of disclosing to the partner (fear of relationship break), suspicion over the importance of condoms (lack of confidence in condoms), lack of knowledge on the importance of condoms after having tested positive.

A 42-year-old-man living with HIV positive steady partner said *"stigma and discrimination still exist in the community, for example there are cases that people do not want to come your home if you are HIV positive. As a result of this, people fear to disclose themselves even to their partner, so they can have sex without condom even if she/he knew herself /himself*.

Another 28-year-old married lady mentioned that *"People hate condoms and are not interested to use them"*.

## Discussion

The issues related to sexual practices, especially the risky one, among HIV-positive individuals are complicated and difficult to give full explanation [1, 5, 18, 19]. Different factors that likely force them to engage in risky sexual practices were reported. For instance, if someone does not believe that the test result is accurate, he or she forms a representation of denial in his or her mind. In this case, the person would fail to change his/her sexual behavior. On the other hand, someone may not have a choice as to when and how to have sex, so he or she depends financially on the corresponding sexual partner. In this case, whatever the person may learn about safe sex, but it is irrelevant to him or her. Furthermore, a person may accept his or her positive sero-status but he or she may have other desires like to have a baby [20]. Conflict findings have been reported weather the improved quality of life due to ART would result in an increase or decrease of sexual desire and risky sexual practices [2, 11, 13].

Most of the time after testing HIV-positive, these individuals remains sexually active, but they experience the sexual adjustment period during which they reported feeling less comfortable with sex lives [21].These issues were raised significantly in the current study. Some of the study participants claimed a decline in sexual desire promptly after testing positives and had sexual intercourse only for sake of their partners need in order to maintain their relationship.

Almost all interviewees reported that they had been experiencing multiple sexual partnerships and most of them had not been using condom at all or inconsistently using them before testing positives. Interestingly, despite their previous history of experiencing multiple sexual partnerships, all the interviewees in this study reported single sexual partnership after testing positive. In contrary to these, previous quantitative studies indicated multiple sexual partnership practices were common among HIV-positives [14, 22]. These variations might be attributed a small sample size and the effect of social desirability bias as the interviewees were face to face. Disclosure of sero-status to partner is a big issue in the time of HIV pandemic [8, 21, 23]. Most of the interviewees in this study were able to know their partner sero-status, though there were claims some had suspicion over their partner serostatus despite they were told orally, but were not willing to be tested together. This study revealed that partners' disgustful expressions and discouragement in sexual relationship were common.

Very important area that needs emphasis is the sexual practices among sero-discordant partners. Sero-discordance partnership had been reported. Among those who were discordant though some had reached on consensual agreement and used condom there were reported disagreements to have safer sex with their sero-discordant partners that they forced to engage in risky sexual practices. The other dimension related to sexual practices among these groups is the condom use pattern. Some of interviewees had used condoms consistently, had frequent discussion and reach on consensual agreement to have safe sex with their partners. On the other hand, there were also individuals who did not use or inconsistently used condoms because of the partners' disagreement and lack of clear knowledge on the importance of condom after testing positive with concordant partner. These are the areas that critically warrant emphasis in the future interventions. The existence of beliefs that his/her partner also sero-positive and was not entitled to use condom were important issues raised. Equipping with clear knowledge on the importance of using condom after testing positives very important way of just encouraging the use of condom consistently.

It was figured out that majority were capable to mention after testing positive condom use would prevent from unwanted pregnancy, but there were inconsistencies in respect to the use of condoms in protecting from re-infection with other strain of viruses that potentially results to drug resistance and have negative impact on their future health status. Even there were consistent condom users but unable to identify the importance of condom use to reduce re-infections. In general why the HIV-positives fails to use condom, the interviewee pointed out that these could be due to the fear of disclosure, lack of clear knowledge on the importance of using condom after testing positive. The suspicion over the efficacy of the condom in protecting against the transmission was also raised.

## Conclusion

Very important issues on sexual practices of HIV-positives were elicited. Fail to reach on consensual agreement on condom use, lack of clear knowledge on the importance of condom in a condition where both partners' sero-positive and fear of sero-status disclosure were important reasons raised for not using condoms. Very importantly, equipping with a skill to negotiate on condom use and providing them with clear information on the importance consistent use of condom to prevent re-infections and encouraging sero-status disclosure are important. Furthermore, the issue of sero discordances should not be over looked. Some sexual practices might be underestimated due to a small number of the interviewees included that the findings of this study need to be interpreted cautiously.
